# Spatial–Numerical Magnitude Estimation Mediates Early Sex Differences in the Use of Advanced Arithmetic Strategies

**DOI:** 10.3390/jintelligence11050097

**Published:** 2023-05-18

**Authors:** Marina Vasilyeva, Elida V. Laski, Beth M. Casey, Linxi Lu, Muanjing Wang, Hyun Young Cho

**Affiliations:** Lynch School of Education and Human Development, Boston College, Chestnut Hill, MA 02467, USA; laski@bc.edu (E.V.L.); caseyb@bc.edu (B.M.C.); lulk@bc.edu (L.L.); wangadx@bc.edu (M.W.); choql@bc.edu (H.Y.C.)

**Keywords:** numerical magnitude, number line, spatial–numerical, arithmetic strategy, sex differences

## Abstract

An accumulating body of literature points to a link between spatial reasoning and mathematics learning. The present study contributes to this line of research by investigating sex differences both in spatial representations of magnitude and in the use of arithmetic strategies, as well as the relation between the two. To test the hypothesis that sex differences in spatial–numerical magnitude knowledge mediate sex differences in the use of advanced strategies (retrieval and decomposition), two studies were conducted. Study 1 included 96 US first graders (53% girls); Study 2 included 210 Russian first graders (49% girls). All participants completed a number line estimation task (a spatially based measure of numerical magnitude knowledge) and an arithmetic strategy task (a measure of strategy choice). The studies showed parallel results: boys produced more accurate numerical magnitude estimates on the number line estimation task and used advanced strategies more frequently on the arithmetic task. Critically, both studies provide support for the mediation hypothesis (although there were some differences in the pattern obtained for the two strategies). The results are discussed in the context of broader research about the relation between spatial and mathematical skills.

## 1. Introduction

Early mathematics skills are among the strongest predictors of academic achievement and broader life success, more so than reading skills (Duncan et al. 2007; National Mathematics Advisory Panel 2008). Thus, differences in early math skills may have substantial implications for later achievement. Around first grade, sex differences favoring boys begin to emerge in some, but not all, areas of mathematics (Fischer and Thierry 2022; Hyde et al. 2008; Lindberg et al. 2010). In fact, a recent investigation that examined multiple numerical skills in children 6–13 years of age concluded that boys’ advantage is the exception rather than a rule (Hutchison et al. 2019). In light of this evidence, the best way to advance actionable knowledge about sex differences in mathematics is to focus on the skills that comprise the exception. One notable exception is boys’ advantage in early arithmetic, especially in the use of more advanced arithmetic strategies. Among several factors that may contribute to this sex difference in early strategy use, sex-based variability in spatial reasoning is a particularly likely candidate. 

A substantial body of research has documented that (a) spatial skills are strongly predictive of math skills, including arithmetic problem solving (Gunderson et al. 2012; Hawes et al. 2019; Mix and Cheng 2012; Mix 2019; Wai et al. 2009; Xie et al. 2020); (b) boys tend to have an advantage in spatial skills (Voyer et al. 1995; Levine et al. 2016); and (c) boys engage spatial reasoning to a greater extent when solving math problems (Geary et al. 2023). The primary emphasis of this work has been on identifying the role of general spatial reasoning, using tasks such as mental rotation, in children’s math performance. A complementary approach is to focus on measures that link spatial reasoning and numerical knowledge, which may illuminate more proximal mechanisms of sex differences in arithmetic problem solving. A number line estimation task is such a measure of spatial–numerical knowledge; it captures numerical magnitude understanding by mapping it onto spatial magnitude (i.e., distance). This task has consistently revealed sex differences whereby boys produce more accurate numerical estimates than girls (Gunderson et al. 2012; Hansen et al. 2015; Hutchison et al. 2019; LeFevre et al. 2010; Reinert et al. 2017; Thompson and Opfer 2008; Tian et al. 2022). In the present study, we consider sex differences in spatial–numerical knowledge, rather than in general spatial skills, in relation to arithmetic problem solving.

Most investigations that have examined the association between spatial–numerical knowledge and arithmetic utilized measures of accuracy, rather than strategy choice. Yet, there is growing recognition of the contribution of arithmetic strategies; the way children solve problems has been found to be more predictive of later mathematics achievement than their accuracy (Geary 2011; Torbeyns et al. 2005). Furthermore, sex differences in arithmetic strategy use are even larger than concurrent sex differences in accuracy (Casey and Ganley 2021). The present study tested the hypothesis that boys’ advantage in spatial–numerical knowledge might be a possible mediational mechanism for sex differences found in advanced arithmetic strategy use among first graders.

### 1.1. Arithmetic Strategies

In a prominent developmental theory (The Overlapping Waves Theory, Siegler 1996), cognitive development is characterized by the acquisition of new problem-solving strategies and increasingly adaptive choice among available strategies in ways that maximize efficiency and accuracy. Within this theory, arithmetic development is marked by greater use of more advanced strategies over time. Children initially solve arithmetic problems using counting—first with fingers and later mentally (Secada et al. 1983; Siegler and Robinson 1982). Yet, counting strategies are laborious and error-prone, particularly when problems involve multi-digit numbers. Therefore, it is essential for children to move toward using more advanced arithmetic strategies—retrieval and decomposition (Geary et al. 2004; Siegler 1987). Retrieval refers to the recall of memorized number facts, which works effectively until children encounter more complex, unfamiliar problems. Even for adults, memorizing number facts beyond those involving single digits would put an unnecessary burden on the memory system. So, for children to engage in more complex problem solving they must acquire a new strategy. 

Decomposition, sometimes referred to as derived math fact strategies (Dowker 2014) or fact-utilizing strategies (Gaidoschik 2012), is considered one of the most advanced arithmetic strategies because it has several advantages over other computational approaches. This approach involves problem solving where children use arithmetic facts they already know to solve unfamiliar problems. For example, a child asked to solve 6 + 5, may think, “I know 5 + 5 = 10, so 6 + 5 must be one more than 10, so the answer to 6 + 5 is 11.” This strategy allows children to mentally figure out the answer to problems they did not know how to solve previously, including mixed and double-digit problems, and can be applied to a wide range of problems (Ashcraft and Stazyk 1981). Furthermore, it requires active problem solving where children manipulate the initial numbers to transform them (Baroody and Dowker 2003). Thus, it provides an early practice ground for thinking about numbers in flexible ways that extend to later mathematics. It is, therefore, not surprising that early use of decomposition predicts math skills concurrently and longitudinally (Carr et al. 2008; Casey et al. 2015; Casey et al. 2017; Geary 2011; Geary et al. 2004). A longitudinal study showed that the use of decomposition in first grade was the key factor predicting math competency through middle school (Geary et al. 2023).

### 1.2. Sex Differences in Arithmetic Strategies

There is evidence of sex-related variability in the use of arithmetic strategies starting in early elementary school. In particular, boys use retrieval and decomposition more frequently than girls (Carr et al. 2008; Geary 2011; Geary et al. 2004; Imbo and Vandierendonck 2007). Instead, girls generally are more likely than boys to use the counting strategy (Carr and Davis 2001; Carr and Jessup 1997; Fennema et al. 1998). This pattern of sex differences may have implications for girls’ later math achievement. A longitudinal investigation showed that greater frequency of using decomposition in first-grade girls was associated with higher math reasoning skills four years later (Casey et al. 2015). 

With an increasing recognition of the importance of early arithmetic strategies for subsequent math learning, the phenomenon of sex differences in strategy use needs to be explored in greater depth, especially considering some limitations of the current literature. First, in existing studies, advanced strategies are often lumped together. For example, several studies that found a male advantage in the use of advanced strategies combined decomposition into a single category with retrieval and/or mental counting (Gaidoschik 2012; Carr and Alexeev 2011; Carr and Davis 2001). Given the unique role of decomposition in predicting math learning, it is important to examine sex differences specifically in decomposition, separate from other types of advanced strategies. One recent study that analyzed each strategy separately did report sex differences favoring boys in the use of decomposition among Danish elementary school students (Sunde et al. 2019). Yet, this study included only single-digit arithmetic problems; further work would have to include a broader range of problems to achieve a more comprehensive view of sex-related variability in strategy choice. 

Another way to extend the current literature is to go beyond documenting a sex difference in strategy use toward a greater understanding of potential antecedents of this phenomenon. What could account for a greater tendency in boys, compared to girls, to select more advanced strategies (i.e., retrieval and decomposition) among other possible approaches (i.e., counting) to solving arithmetic problems? We propose that it may be boys’ advantage in spatial–numerical skills that contributes to their greater use of advanced problem-solving strategies when doing early arithmetic. Specifically, we hypothesize that sex differences in children’s choice of advanced strategies may stem from the relation between spatially based number magnitude knowledge and strategy use. 

### 1.3. Potential Influence of Numerical Magnitude Estimation on Arithmetic Strategies 

There is a general consensus that numerical magnitude knowledge is foundational for math learning (Schneider et al. 2017; Siegler and Opfer 2003). Key theories of numerical cognition propose that numerical magnitude is represented spatially in the mind, along a mental number line (Case et al. 1996; Dehaene 2011; Siegler and Lortie-Forgues 2014; Sowder 1992). Such representations become more precise with age and experience, increasingly reflecting the interval property of the number system (Berteletti et al. 2010; Laski and Siegler 2007; Siegler and Opfer 2003). More accurate spatial–numerical representations can be expected to facilitate performance across a variety of numerical tasks by providing cues about distance between numbers. 

In the domain of arithmetic specifically, numerical magnitude knowledge has been theorized to facilitate problem solving by constraining the search space for answer choices (Booth and Siegler 2008; Laski and Yu 2014). In children’s initial attempts to use retrieval, they invariably produce both correct and incorrect responses. Children with a better understanding of relative numerical magnitudes should be more likely to select among answers that would be plausible given the magnitudes of the addends (e.g., 8 + 4 cannot make 6 or 30), than to search the full range of known numbers. This constrained search space should lead to a greater probability of retrieving a correct response to a given problem, which, in turn, leads to a stronger memory association between the problem and its answer (Shrager and Siegler 1998). Once the association is established with the support of numerical magnitude knowledge, retrieval then becomes an automatic memory process. 

Consistent with this view, children with more accurate mental representations of numerical magnitude generate responses to arithmetic problems that are closer to the actual sum, compared to those with poorer number magnitude knowledge (Booth and Siegler 2008; Geary 2011; Laski et al. 2016; Laski and Yu 2014; Schiffman and Laski 2018; Siegler and Ramani 2009). Furthermore, recent studies provide evidence that children with better numerical magnitude knowledge use retrieval more frequently than those with poorer numerical magnitude knowledge to generate accurate answers to single-digit arithmetic problems (Vanbinst et al. 2015).

Because magnitude knowledge limits the “search space” for accurate responses, we posit that it can facilitate not only retrieval, but also a decomposition strategy. To our knowledge, there are no studies establishing a relation between numerical magnitude knowledge and decomposition. Yet, if numerical magnitude knowledge constrains the search for answers at each step of executing decomposition (e.g., decomposing single-digit addends or combining the tens and ones in a multi-digit number), it should have a similar or even greater effect on the use of decomposition strategies. 

Furthermore, there are additional ways in which mental representations of numerical magnitude may have a particular effect on the execution of the decomposition strategy. One is that it offers a framework for moving along a spatial continuum when breaking down and recombining numbers. This possibility is supported by evidence suggesting that people rely on the mental number line to increase or decrease numeric magnitude when adding or subtracting. In particular, eye-tracking studies show that during mental arithmetic, individuals’ visual attention shifts as though moving along a number line—to the right when doing addition and to the left when doing subtraction (Knops et al. 2009; Pinhas and Fischer 2008). Another is that because spatial representations of numerical magnitude have been shown to aid children’s memory for numbers (Thompson and Siegler 2010), this should allow for more efficient use of decomposition as it is necessary to hold intermediate information in mind when executing a multi-step strategy. 

In contrast to the use of advanced strategies, there is no reason to expect that numerical magnitude knowledge promotes the use of counting. A counting strategy can be executed by relying on the ordinal sequence of numbers (e.g., 8 + 4 is…9, 10, 11, 12). Children are able to recite the counting string before they can accurately represent the magnitude of those numbers (Siegler and Lortie-Forgues 2014). If students know the counting sequence, they do not need to rely on numerical magnitude knowledge (e.g., understand the relative magnitude of the addends and the sum) to generate an accurate answer. 

In sum, there are multiple reasons to expect that spatial–numerical magnitude knowledge influences children’s arithmetic strategy choice. Strategy choice models indicate that children select which strategy to use on a given problem in order to maximize efficiency (Shrager and Siegler 1998). Numerical magnitude knowledge is expected to make it easier to execute retrieval and decomposition. Therefore, children with more accurate spatial–numerical magnitude representations can be expected to use these strategies more frequently. On the other hand, numerical magnitude knowledge is not expected to confer an advantage on the use of counting; thus, it is unlikely to be related to the frequency with which children use this strategy. 

### 1.4. Purpose of the Present Study

The review of extant research indicates that there is a sex difference favoring boys in terms of the frequency of using retrieval and decomposition strategies, as well as the number line task tapping the knowledge of symbolic numerical magnitude. Given the mechanisms proposed above, whereby the use of retrieval and decomposition is facilitated by spatially based numerical magnitude knowledge, we hypothesized that a sex difference in the frequency of using these strategies is mediated, at least in part, by sex differences in number magnitude representations. In contrast, we expect no mediation of sex differences in the use of counting strategies via numerical magnitude knowledge because this strategy can be executed using knowledge of the count sequence alone. 

We tested these hypotheses with first-grade students after children had received some instruction but were still in the early stages of learning arithmetic. Two studies were conducted that included students from two different cultural/educational contexts: the US (Study 1) and Russia (Study 2). In both countries, arithmetic problem solving is a focus of instruction in first-grade classrooms, beginning with single-digit problems with sums of 10 or less and moving to problems with a sum crossing 10. Yet, there are certain differences between these contexts. In particular, children in Russia enter first grade one year later than in the US and the Russian educational system is more centralized. With respect to the math curricula, while both countries have similar curricular goals and standards, US teachers introduce a variety of approaches to arithmetic problem solving and encourage students to choose among them, whereas Russian teachers place a greater, more explicit, emphasis on the use of advanced strategies. Including participants from different contexts allowed us to examine the generalizability of the effects. 

## 2. Study 1: Method

### 2.1. Participants

The study included 96 first graders (53% girls) from a large US city, with a mean age of 6.98 years (*SD* = .39). Children were recruited from urban schools serving racially/ethnically diverse population (Black: 13–16%, Hispanic: 17–50%, Asian: 5–8%; White: 27–58%), with a large percentage of students from low-income families (48–68%). 

### 2.2. Materials and Procedure

The testing was conducted one-on-one in a quiet room at the child’s school. It included mental arithmetic and number line estimation tasks, with the arithmetic task presented first. 

#### 2.2.1. Arithmetic Task

Participants were presented 12 addition problems, all of which involved crossing 10. The first six were single-digit problems with sums over 10 (e.g., 7 + 9), followed by six mixed-digit problems with sums between 20 and 50 (e.g., 7 + 25, 17 + 4). Each problem was printed on a separate sheet; they were presented one at a time and read aloud by the tester. As the child was solving the problem, the tester recorded any overt signs of strategy use, such as counting out loud or using fingers. When there were no overt behaviors, the tester asked the child, after a response was provided, how they “figured it out.” In addition to written notes, responses were audiotaped. Notes and audio recordings were used to code children’s strategies into one of four categories: counting, retrieval, decomposition, and other. A strategy was coded as decomposition when the child reported solving the problem by breaking it up into two or more problems (e.g., solving 8 + 4 as 8 + 2 = 10 and 10 + 2 = 12; or solving 8 + 7 as 7 + 7 = 14 and 14 + 1 = 15). 

Two types of reliability checks were conducted. To determine the internal consistency of items, we computed KR-20 (Kuder–Richardson coefficient) for accuracy scores; this measure of reliability is used with binary outcomes (correct/incorrect). Both single- and mixed-digit problems exhibited good reliability with KR-20 values of 0.83 and 0.87, respectively To determine the inter-rater reliability of strategy coding, data from 20% of the sample was coded independently by two raters. Their agreement rate was 96%. All cases of disagreement were resolved in consultation with other researchers. 

#### 2.2.2. Number Line Estimation

Participants were read a numeral and asked to mark its position on a number line with only the endpoints (0 and 100) labeled. Previous estimates were not visible on later trials. Following two practice trials in which children were asked to indicate the positions of 0 and 100 and shown their location if needed, children were presented with 22 test trials without feedback. The numbers presented were 2, 3, 5, 8, 12, 17, 21, 26, 34, 39, 42, 46, 54, 58, 61, 67, 73, 78, 82, 89, 92, and 97. A different random order of the numbers was generated for each child. The measure of children’s accuracy was calculated as the average percent absolute error: PAE = (|child’s estimate − estimated quantity|/scale) × 100.

## 3. Study 1: Results and Discussion

Prior to conducting the main analysis, we compared performance on single- and mixed-digit addition problems. The two types of problems were highly correlated both in terms of accuracy and frequency of using specific strategies (correlations varied between *r* = .75 and *r* = .89, all *p* < .001). Furthermore, these problems showed a parallel pattern of sex findings (i.e., there was no significant Sex x Problem interaction for any strategy examined, all *p* > .05). Thus, the two problem types were combined in subsequent analyses. 

Descriptive statistics are presented in Table 1. As expected based on prior research, there was a sex difference in the number line estimation task—boys, on average, had a smaller percent absolute error (PAE) than girls. With respect to strategy use, the pattern varied depending on strategy. Decomposition revealed clear sex differences—boys chose it more often than girls. Retrieval was used very rarely and, although boys tended to use it more often than girls, sex differences were marginal. In contrast, counting was the most frequently used strategy, and girls used it significantly more often than boys. 

Next, we examined correlations among children’s accuracy on the number line estimation task and the frequency of different strategies (see Table 2). Percent absolute error on the number line was negatively correlated with the frequency of both decomposition and retrieval strategies—that is, children who generated more accurate number line estimates tended to use these strategies more often. In contrast, there was a marginal positive correlation between number line absolute error and the use of counting strategies. 

**Figure 1:**
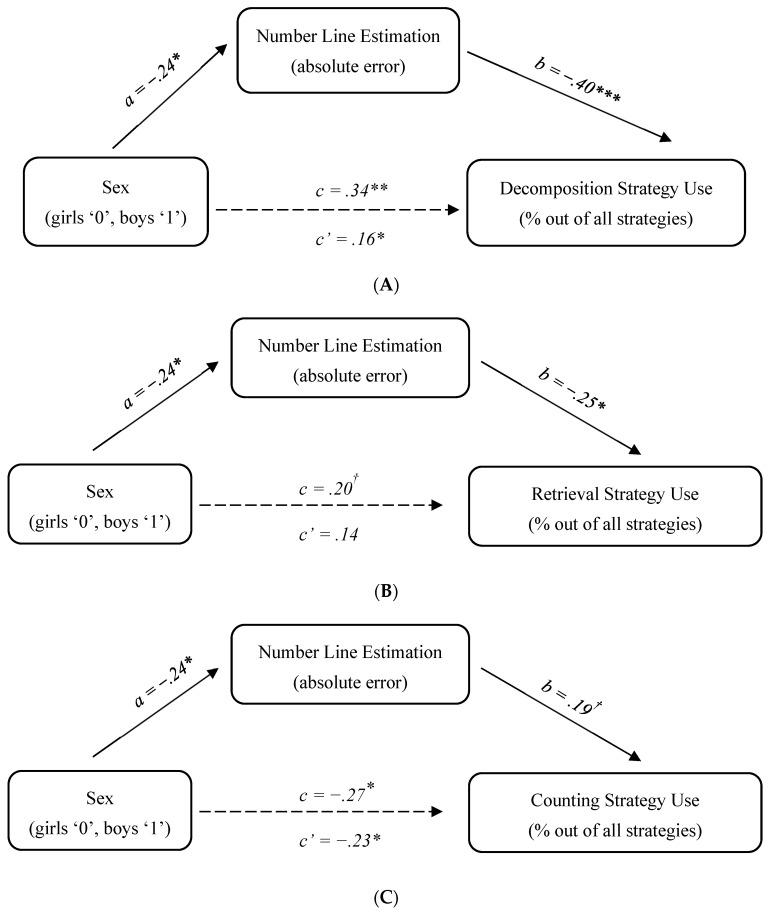
Study 1 mediation analysis: (**A**) decomposition; (**B**) retrieval; (**C**) counting. Note. Standardized coefficients are reported in all models; ^†^ *p* < .1, * p < .05, ** p < .01, *** p < .001.

In the final stage of analysis, we determined to what extent the observed sex differences in the number line estimation task accounted for sex differences in the key strategies examined: decomposition, retrieval and counting. Although sex differences in retrieval were marginal, there was still a possibility that the indirect path between sex and retrieval frequency via number line estimation would be significant (Hayes 2017). Thus, for each strategy we tested the same model, controlling for the child’s age: sex -> number line estimation error-> strategy frequency. The models were tested by conducting a bias-corrected bootstrapping mediation analysis (Dearing and Hamilton 2006), using the SPSS macro “PROCESS” (Preacher and Hayes 2004). The results are shown in Figure 1; note that no overall model fit indices are reported given that the models are just-identified. 

For decomposition, the results show a significant indirect effect of sex on the frequency of this strategy via number line estimation: 95% CI [−8.24; −.93]. As for the direct effect of sex on decomposition use, it was highly significant before the mediator was entered in the model (*p* < .001); once the mediator was added, the direct effect decreased but remained significant (*p* = .02), indicating partial mediation (see Figure 1A). Because the mediator and the outcome were measured in this study concurrently, we followed-up on this finding by testing an alternative model with decomposition frequency as the mediator and number line error as the outcome. In the alternative model, the indirect effect was not significant: 95% CI [−.008; .029]. The pattern of findings thus supports the hypothesized role of number line estimation as a mediator of sex differences in decomposition frequency. 

Similar to decomposition, there was a significant indirect effect of sex on the frequency of retrieval via number line estimation: 95% CI [−1.19; −.09]. The direct effect of sex on retrieval use was marginal before the mediator was added in the model (*p* = .057) and further decreased after the mediator was entered *(p* = .161). This pattern of findings shows that there is a significant indirect effect of sex on retrieval frequency via number line estimation in the absence of a direct effect (see Figure 1B). An alternative model (with retrieval as the mediator and number line error as the outcome) showed that the indirect effect of sex on number line estimation via retrieval use was not significant: 95% CI [−.0001; .014].

With regard to counting, the use of this strategy was characterized by sex differences; notably, they were in the opposite direction compared to decomposition and retrieval. For consistency, we tested whether there was also an indirect path between sex differences in this strategy use and the number line estimation accuracy. The results of the mediation analysis show that while there was indeed a significant direct effect of sex on counting frequency (95% CI [2.15; 30.67]), the indirect effect was not significant (95% CI [−1.16; 6.30]). In other words, a higher frequency of the counting strategy in girls, compared to boys, was not mediated by their number line estimation error (see Figure 1C). 

In sum, the results of Study 1 provide initial support for our hypotheses, demonstrating a significant indirect path from child’s sex to the use of retrieval and decomposition strategies via numerical magnitude knowledge. The observed pattern was unique to the advanced arithmetic strategies—it did not extend to the use of the counting strategy. Given the novelty of these findings, we aimed to determine their generalizability. Thus, we conducted Study 2 with a sample of students from a different cultural and instructional context. Furthermore, in Study 2 we aimed to provide a more rigorous test of our mediation hypothesis by controlling for participants’ general intelligence and by assessing the outcomes six months after assessing predictor measures.

## 4. Study 2: Method

### 4.1. Participants

The study included 210 first graders (49% girls) from a large Russian city. At the first testing session (fall of 1st grade), the mean age was 7.28 years (*SD* = .33); the time between the two testing sessions was, on average, six months. Participants were recruited from municipal (public) schools. As reported by parents, they represented diverse socio-economic groups, with the educational levels varying as follows: 25% high-school diploma with or without vocational training; 16% some college education; 48% college degree; and 11% graduate training. 

### 4.2. Materials and Procedure

Testing was conducted as in Study 1 with two exceptions. First, Study 2 included a Raven’s task, which was added to control for general non-verbal intelligence. Second, whereas all assessments in Study 1 were conducted in the spring of first grade, in Study 2 the number line and Raven’s tasks were administered at the start of first grade, but the arithmetic task was administered at the end of the school year. 

#### 4.2.1. Number Line Estimation 

This task was identical to that in Study 1. 

#### 4.2.2. Arithmetic Task

This task was parallel to that in Study 1, except that the number of items was increased to 16: eight single-digit problems with sums over 10 and eight mixed-digit problems with sums between 20 and 50. The coding and reliability procedures were parallel to those in Study 1. Both single- and mixed-digit problems exhibited good reliability with KR-20 values of 0.90 and 0.89, respectively. The interrater agreement rate was 96%. 

#### 4.2.3. Raven’s Matrices

We used Raven’s colored progressive matrices designed to estimate non-verbal intelligence in children 5 through 11 years old (Raven and Court 1998). The child received a booklet with 36 items printed on separate pages. On each page, a geometric pattern with a missing piece was depicted at the top, with six answer choices depicted below. The task was to select the answer choice that would fill in the missing piece. Two practice trials were administered with feedback. On test trials, the child selected one of the choices with no feedback. The task duration was 15–20 min. The score was calculated as the percentage of correctly solved items. 

## 5. Study 2: Results

As in Study 1, children’s performance on single- and mixed-digit addition problems was correlated both in terms of accuracy and the frequency of using specific strategies (correlations varied between *r* = .79 and *r* = .89, *p* < .001), and the two types of problems were combined in subsequent analysis. Descriptive statistics are presented in Table 3. Note that there was no effect of sex on Raven’s task, indicating that any sex differences observed in other tasks could not be due to general cognitive differences between boys and girls. 

Performance on the number line task was similar to that observed in Study 1—mean percent absolute error for the whole sample was the same in the two studies, with boys producing more accurate estimates than girls. Additionally, parallel to Study 1 was the pattern of sex-related variability in the frequency of specific strategies. In the case of decomposition and retrieval, boys, on average, used these strategies more frequently than girls, whereas in the case of counting, sex differences showed the opposite pattern. In Study 2, the sex differences in the use of retrieval reached statistical significance (*p* = .015), whereas in Study 1 they did not (*p* = .057). Two other differences in the results of the two studies are noteworthy. In particular, compared to Study 1, the frequencies of decomposition and retrieval strategies were notably higher. Furthermore, the accuracy of arithmetic problem solving among the Russian participants was not only higher than that of the US participants in Study 1, but also it showed a significant sex difference favoring boys. 

To better understand the dissimilarity in the pattern of accuracy findings across the two studies, we ran a follow-up analysis with a subgroup of Russian students that used decomposition with a similar frequency as the US students in Study 1. This analysis allowed us to examine whether the discrepancy in accuracy results might be related to differences in the level of strategy use. To conduct this analysis, we ordered all Study 2 participants (*N* = 210) according to their strategy frequency and then eliminated the top 20%, resulting in a subsample (*N* = 168) that was relatively equally divided between boys (N_b_ = 82) and girls (N_g_ = 86) and whose overall level of decomposition use (13%) was comparable to that in the US sample (14%). The analysis of this subsample showed that boys still outperformed girls in terms of decomposition strategy use (*M_b_* = 17%, *M_g_* = 10%, *t*(166) = 2.70, *p* = .008), but the sex difference in accuracy was not significant (*M_b_* = 69%, *M_g_* = 65%, *t*(166) = 1.82, *p* = .07). Thus, in the Russian sample, students at the lower levels of decomposition use showed sex differences in strategy frequency but not in accuracy, which is parallel to Study 1 results.

Correlations among percent absolute error in the number line task and the frequency of different strategies are presented in Table 4. As in Study 1, the average absolute error of number line estimation was negatively correlated with children’s use of retrieval and decomposition strategies. That is, the children who generated more accurate number line estimates tended to use these advanced strategies more often. In contrast, the correlation between number line absolute error and use of the counting strategy was positive.

To test the mediation hypothesis, we conducted the same analyses as in Study 1, using the SPSS macro “PROCESS” created by Preacher and Hayes (2004). The model tested was as follows, controlling for age and Raven’s scores: sex -> number line estimation error -> strategy frequency. Because children’s accuracy in the number line task was measured six months before the assessment of their arithmetic strategies, we did not consider alternative models with strategy frequency as a potential mediator of sex differences in the number line estimation. 

**Figure 2:**
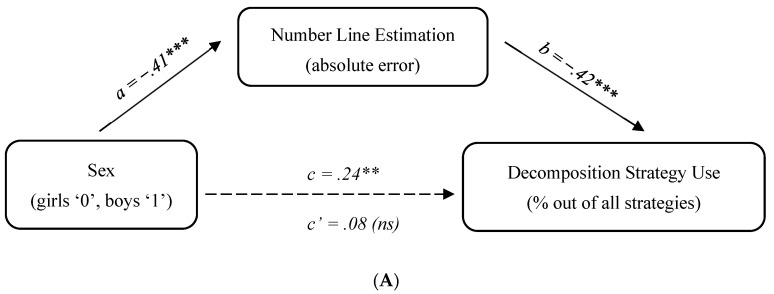

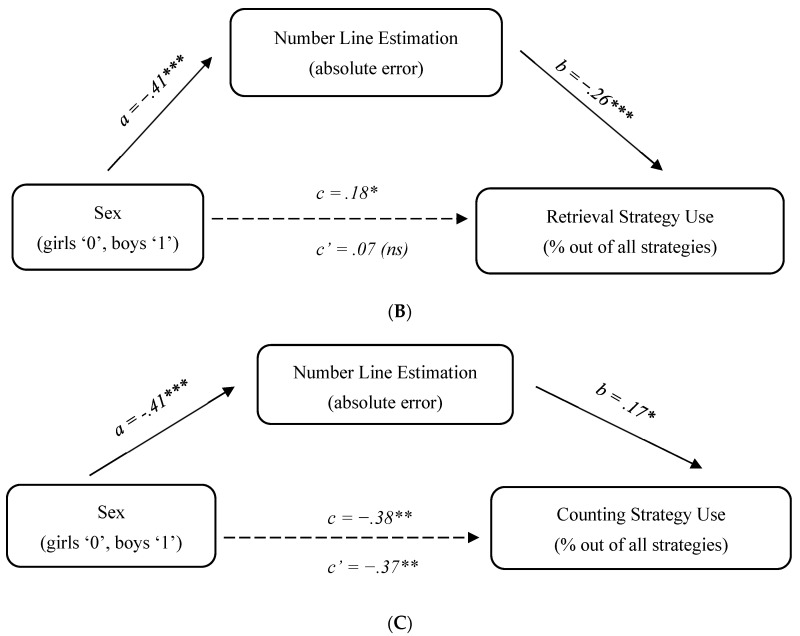
Study 2 mediation analysis: (**A**) decomposition; (**B**) retrieval; (**C**) counting. Note. Standardized coefficients are reported; ^†^ *p* < .1, * p < .05, ** p < .01, *** p < .001.

The results for retrieval and decomposition showed a similar pattern, indicating a full mediation for both strategies. Specifically, the direct effect of sex on strategy frequency was significant prior to entering the mediator into the model, but became insignificant once the mediator was added: decomposition (from *p* < .001 to *p* = .248) and retrieval (from *p* = .014 to *p* = .309). The indirect path from sex to strategy frequency via number line absolute error was significant in both cases: decomposition, 95% CI [−.13; −.06], and retrieval, 95% CI [−.04; −.01]. Thus, sex differences in number line estimation accounted for sex differences in both decomposition and retrieval strategies (see Figure 2A,B).

With respect to counting, the opposite pattern was observed. The direct effect of sex on counting use was significant both before and after the mediator was entered in the model (for both, *p* < .001), whereas the indirect path was not significant, 95% CI [−.03; .04]. In other words, sex differences in the use of the counting strategy could not be accounted for by number line estimation (see Figure 2C). 

## 6. General Discussion

Questions concerning the nature of the relation between spatial reasoning and mathematics learning have attracted the attention of both scientists and educators. Investigating the mechanisms underlying sex differences in these aspects of cognition may contribute to a better understanding of this relation, which in turn may have far-reaching consequences for broadening participation in STEM disciplines. The current results demonstrate the generality of sex differences in spatial–numerical magnitude knowledge and the potential contribution of these differences in explaining sex variability in early arithmetic. As expected, boys used advanced arithmetic strategies more often than girls and these sex differences were mediated by spatial–numerical magnitude knowledge; on the other hand, sex differences in counting favored girls and were not accounted for by spatial–numerical magnitude knowledge. In this concluding section, we situate the findings within a larger context of extant research and consider their implications for development and instruction. 

### 6.1. Sex Differences in Spatial–Numerical Magnitude Knowledge and Arithmetic Strategy Use

Prior research documented sex differences in spatial–numerical magnitude knowledge among US children. More specifically, studies have found a male advantage in number line estimation (Hansen et al. 2015; Hutchison et al. 2019; Tian et al. 2022). The current results demonstrate that this phenomenon generalizes to other cultural contexts—Russian boys produced more accurate number line estimates than girls, just like their US peers. This observed sex difference in number line estimation is likely connected to broader differences in spatial reasoning. In fact, spatial skills have been found to predict the accuracy of spatial representations of numerical magnitude (Gunderson et al. 2012). Furthermore, a recent investigation showed that a male advantage in the number line estimation task was associated with a parallel advantage in “purely” spatial tasks (Tian et al. 2022). Specifically, mental rotation and scaling—the two skills that produce the most robust evidence of sex differences in spatial reasoning (Levine et al. 2016; Voyer et al. 1995)—accounted for a significant proportion of sex-related variability in number line estimation. 

The current results also demonstrate sex differences in arithmetic strategy use, consistent with prior research (Casey and Fell 2018; Geary 2011; Geary et al. 2004; Imbo and Vandierendonck 2007; Sunde et al. 2019). That is, both US and Russian girls tended to use counting on a greater number of problems than boys, while boys were more likely to use retrieval and decomposition than girls. Whereas the direction of sex-based variability was the same across the two studies, several differences were observed in the use of advanced strategies. In particular, with respect to retrieval, the sex difference was significant among Russian students, but only marginal among US students. The overall use of this strategy was noticeably different across the two studies: 2.4% in Study 1 and 11% in Study 2. Thus, our ability to find sex differences in the use of retrieval in Study 1 may have been constrained by the overall low frequency of this strategy among the US students. 

With respect to decomposition, while a sex difference, favoring males, was generalized across the two studies, there were notable differences in the overall use of this strategy, with the US students using it less frequently. The reason why the overall frequency of decomposition (as well as retrieval) varied across the two contexts is not clear. It may be due in part to differences in the demographic characteristics of the participants; the US sample was primarily low-income, while the Russian sample was more socioeconomically diverse. These background differences may relate to differences in experiences with arithmetic. Another possible explanation is that the frequency of decomposition use reflects the extent to which it is emphasized in school instruction. Our informal observations in classrooms revealed distinct approaches to arithmetic strategy instruction in the two contexts. US teachers tended to encourage students to try out different strategies, whereas Russian teachers tended to be more direct in steering students away from counting toward more advanced strategies. While the current data do not allow us to test these potential explanations, they could be pursued in further research. Whatever the reasons are for the variability in decomposition frequency across the two samples, it is notable that both demonstrated significant sex differences in the use of this strategy. This finding suggests that sex differences persist across different levels of strategy use. 

Although the main focus of the present study was on strategy use, we also obtained data on arithmetic accuracy and its examination revealed an intriguing difference across the two studies. In Study 2, Russian boys’ higher frequency of using decomposition was also accompanied by higher accuracy compared to girls, but in Study 1 (where students used decomposition less frequently), boys used decomposition more frequently than girls but there was no corresponding accuracy advantage. Generally, greater use of decomposition is associated with more accurate problem-solving, both in the short term and in predicting later math achievement (Casey et al. 2017; Geary et al. 2004; Vasilyeva et al. 2015). Yet, despite the sex differences in decomposition use in Study 1, the accuracy was statistically equivalent for boys and girls. 

Comparing the accuracy results across the two studies raises the possibility of a threshold effect—in order for advanced strategies to confer advantages in accuracy, children need to reach a certain level of using them. For example, when the use of decomposition is still relatively infrequent, even though boys use this strategy more often than girls, sex differences may not be noticeable in terms of accuracy. Children may still have difficulty executing the strategy correctly or, even if they execute it correctly, it may have a limited effect on overall accuracy when used on only a small portion of problems. In other words, at lower levels of frequency of advanced strategies, sex differences in strategy use may not translate to differences in accuracy, but with a more frequent use of these strategies, they may have a more significant impact on accuracy. Indeed, consistent with this possibility, in previous research (Shen et al. 2016), US first graders from high-income backgrounds not only demonstrated higher levels of using decomposition than those US students in the present Study 1, but also showed sex differences favoring boys in both strategy and accuracy—a finding parallel to Study 2 with Russian children. 

### 6.2. Spatial–Numerical Magnitude Knowledge and as a Mediator of Sex Differences in Advanced Strategy Use

The central question of the present study was as follows: Given that boys and girls receive the same instruction, *why* do boys use advanced strategies more often than girls? We hypothesized that boys’ more accurate spatial representations of numerical magnitude would facilitate their use of retrieval and decomposition. We posited that spatial–numerical magnitude knowledge constrains the search for plausible answers, aids memory for answers at the intermediary steps of decomposition, and offers a framework (i.e., the spatial mental number line) for breaking down and recombining numbers. Together, these mechanisms were expected to increase the efficiency of retrieval and decomposition, and, thus, according to strategy choice models, lead boys to use these strategies more frequently than girls. If this is the case, then sex differences in the use of advanced strategies should be mediated by spatial–numerical magnitude knowledge. 

Indeed, the current results show a largely parallel pattern of mediation across the two studies. In the case of retrieval, although there were some differences in the extent of the direct effect of sex on strategy use (as discussed above), the indirect path from sex to number line estimation to strategy frequency was significant in both studies. It is this indirect effect via number line estimation (present in both samples) that provides critical support for our hypothesis regarding the role of spatial–numerical magnitude knowledge in the use of retrieval. In the case of decomposition, this knowledge partially mediated the relation between sex and strategy frequency in Study 1 and completely mediated this relation in Study 2. 

There are multiple possible reasons why the mediation would be partial in one study and complete in the other. One is that the sample size in Study 2 was more than twice the size of that in Study 1, increasing the power of statistical analysis. A second is that Study 2 controlled for general cognitive abilities, namely non-verbal intelligence, reducing the variability in the outcome variable to be accounted for by the mediator. Finally, though present in both samples, the sex difference in number line accuracy was greater in Study 2, increasing its potential mediation power. These possible reasons, while speculative, do not undermine the primary finding. Rather, it is noteworthy that whether there was partial or complete mediation, in both studies, sex differences in strategy use were significantly reduced once number line estimation was entered in the model. 

This mediation finding is an important step toward identifying a potential causal relation between spatial representations of numerical magnitude and the use of advanced strategies. The possibility of a causal relation is particularly supported by the results of Study 2 when the mediator variable was measured before the outcome. In other words, the results are consistent with the idea that sex differences in the frequency of using decomposition emerge, at least in part, because of differences in numerical magnitude knowledge. More generally, the results support the view that more accurate spatial representations of numerical magnitude increase the ease with which children are able to execute retrieval and decomposition strategies. 

The relation between spatial–numerical magnitude knowledge and arithmetic strategy use is particularly noteworthy when considered within the larger context of research investigating relationships between spatial and math skills. As noted earlier, several lines of research point to the spatial nature of the number line estimation task. Notably, children with higher levels of spatial skills are likely to produce more accurate estimates of numerical magnitudes (Gunderson et al. 2012), and sex differences favoring boys in spatial skills have been found to mediate sex differences in the number line estimation task (Simms et al. 2016; Tian et al. 2022). In other words, existing research suggests that boys’ advantage in general spatial skills may be in part what leads them to develop a more accurate spatial representation of numerical magnitudes. These findings (sex → spatial skills → spatial–numerical magnitude knowledge) combined with the current results (sex → spatial-spatial numerical magnitude knowledge → advanced arithmetic strategy) raise the possibility that sex differences in spatial skills may have an indirect effect on the use of advanced arithmetic strategies via numerical magnitude knowledge. 

Two other types of evidence that are relevant to interpreting the present results concern the predictors of arithmetic accuracy, the ultimate indicator of math performance. Gunderson et al. (2012) showed that spatial skills predict arithmetic accuracy via number line estimation. Several other studies have shown that arithmetic strategy choice predicts accuracy (Geary 2011; Vasilyeva et al. 2015) and, furthermore, sex differences in strategies mediate sex differences in accuracy (Carr et al. 2008; Shen et al. 2016). Integrating all these lines of research suggests a way in which sex differences in spatial skills may ultimately contribute to variability in arithmetic accuracy through chain mediation: sex → spatial skills → spatial–numerical magnitude knowledge → arithmetic strategy → arithmetic accuracy. It would be worthwhile to test the complete mediation chain in the context of a single study in future research. 

### 6.3. Conclusions and Potential Implications

In sum, the present study suggests a potential mechanism connecting spatial skills to what has been traditionally considered non-spatial arithmetic problem solving. The findings add to the extant knowledge about sex differences in spatial representations of numerical magnitude, as well as arithmetic strategy use, and most importantly capture a mediational relationship between the two for advanced strategies. Combined with previous research, the current findings suggest that the contribution of spatial–numerical magnitude knowledge to advanced strategy use may serve as one of the links connecting general spatial reasoning to arithmetic accuracy. 

While the indirect effect was robust across two studies, it still falls short of causal evidence. It will be important for future research to use experimental interventions to establish causal relations. Furthermore, it would be worthwhile to examine the relation between spatial–numerical magnitude knowledge and advanced strategy use across a broader developmental span. It is possible that the strength of this relation changes with age as children become more experienced and proficient in using these strategies. We suspect that extensive practice will strengthen children’s memory of basic number facts, so that there is a direct and automatic association between a problem and its answer. This may reduce or even eliminate the need to constrain the search space when executing a retrieval strategy, as well as decomposition. 

For the developmental periods when the relation between spatial–numerical knowledge and strategy use is robust, as with the first graders in the present study, future research should investigate what types of spatial magnitude cues may be useful for improving numerical magnitude knowledge and whether the improvements extend to arithmetic strategies. It is likely that using concrete spatial representations of numerical magnitude in the context of arithmetic instruction is an effective approach for facilitating the use of advanced strategies. This approach might help to strengthen the association between abstract number symbols and their corresponding quantity. Many current math curricula already involve the use of materials that contain spatial cues about magnitude that vary with number (e.g., linked unifix cubes where the total length is proportional to number of cubes). However, despite the prevalence of these materials, there has been little systematic investigation of whether the spatial dimensions are more helpful for children’s arithmetic learning than non-spatial materials. In sum, we propose that future research needs to determine whether incorporating materials that provide spatial instantiations of numerical magnitude into strategy training facilitates the use of decomposition via improvements in numerical magnitude knowledge. 

## Figures and Tables

**Table 1:** Descriptive statistics and *t*-test results of sex differences, Study 1.

	Mean_girls_ (SD) *N* = 52	Mean_boys_ (SD) *N* = 44	Mean_overall_ (SD) *N* = 96	*p*-Value (*t*-Test)
Child’s Age (Years), Spring of 1st Grade	6.94 (.06)	7.04 (.05)	6.98 (.39)	.210
Number Line PAE	.15 (.07)	.11 (.06)	.13 (.07)	.019
Decomposition Strategy	5.28 (10.04)	23.48 (35.23)	13.75 (26.70)	.002
Retrieval Strategy	1.44 (3.18)	3.60 (4.28)	2.43 (5.53)	.057
Counting Strategy	77.08 (31.24)	58.34 (37.52)	68.49 (35.34)	.009
Other Strategies	16.19 (29.33)	14.58 (25.11)	15.45 (27.35)	.775
Arithmetic Accuracy % correct out of all items	59.17 (34.67)	61.31 (35.81)	60.15 (35.03)	.768

*Note*: Values reported for each strategy represent the percentage of arithmetic problems (out of all) on which that strategy was used. PAE = (|child’s estimate − estimated quantity|/scale) × 100.

**Table 2:** Correlations between number line estimation and frequency of strategy use, Study 1.

	Number Line PAE	Decomposition	Retrieval	Counting	Other
Number Line PAE	1	−.401 **	−.249 *	.194 ^†^	.187
Decomposition % out of all		1	.021	−.625 **	−.164
Retrieval % out of all			1	−.178	.007
Counting % out of all				1	−.653 **
Other % out of all					1

*Note:* ** *p* < .001, * *p* < .05, ^†^ *p* < .1.

**Table 3:** Descriptive statistics and *t*-test results of sex differences, Study 2.

	Mean_girls_ (SD) N = 103	Mean_boys_ (SD) N = 107	Mean_overall_ (SD) N = 210	*p*-Value (*t*-Test)
Child’s Age (Years), Fall of 1st Grade	7.02 (.36)	7.00 (.33)	7.01 (.34)	.609
Number Line PAE	.16 (.08)	.10 (.04)	.13 (.08)	<.001
Decomposition Strategy	18.38 (22.26)	32.12 (32.93)	25.38 (28.97)	<.001
Retrieval Strategy	8.73 (13.53)	13.46 (14.32)	11.14 (14.11)	.015
Counting Strategy	55.89 (30.44)	31.96 (27.54)	43.70 (31.31)	<.001
Other Strategies	17.02 (25.45)	22.46 (25.12)	19.79 (25.37)	.121
Arithmetic Accuracy % correct out of all items	66.69 (22.20)	75.53 (21.45)	71.23 (22.26)	.003
Raven’s Matrices, % correct out of all items	23.39 (7.18)	23.29 (6.72)	23.34 (6.93)	.926

*Note:* Values reported for each strategy represent the percentage of arithmetic problems (out of all) on which that strategy was used.

**Table 4:** Correlations between number line estimation and strategy use, Study 2.

	Number Line PAE	Decomposition	Retrieval	Counting	Other
Number Line PAE	1	−.422 **	−.263 **	.174 *	.414 **
Decomposition		1	−.074	−.576 **	−.390 **
Retrieval			1	−.303 **	−.097
Counting				1	−406 **
Other					1

*Note*: ** *p* < .001, * *p* < .05.

## Data Availability

The data presented in this study are available on request from the corresponding author. The data are not publicly available due to privacy restrictions.
